# Polyacrylamide Functionalized Graphene Oxide/Alginate Beads for Removing Ciprofloxacin Antibiotics

**DOI:** 10.3390/toxics10020077

**Published:** 2022-02-07

**Authors:** Jung-Weon Choi, Sang-June Choi

**Affiliations:** 1 Green Carbon Catalysis Center, Korea Research Institute of Chemical Technology, 141 Gajeong-Ro, Yuseong, Daejeon 34114, Korea; jwchoi92@krict.re.kr; 2 School of Architectural, Civil, Environmental, and Energy Engineering, Kyungpook National University, Daegu 41566, Korea

**Keywords:** ciprofloxacin, antibiotics, adsorption, graphene oxide, polyacrylamide, alginate, double-network, adsorbent

## Abstract

Ciprofloxacin (CPX), a widely used antibiotic, was removed by synthesizing graphene oxide/calcium alginate–polyacrylamide (GO/Ca-Alg_2_–PAM) beads, a three-dimensional double-network complex. The synthesis of GO/Ca-Alg_2_–PAM beads was performed by crosslinking and cation exchange mechanisms with graphene oxide (GO), sodium alginate (Na-Alg), and polyacrylamide (PAM). The properties of GO/Ca-Alg_2_–PAM beads were confirmed using field emission scanning electron microscopy, Fourier transform infrared spectroscopy, and a thermogravimetric analysis. Furthermore, isothermal adsorption experiments were performed and fitted using three isothermal adsorption models (Langmuir, Freundlich, and Temkin). The adsorption isotherm experimental data fit well with the Langmuir isotherm model with a *q_m_* value of 6.846 mg/g. In addition, the spontaneous reaction of the CPX adsorption using GO/Ca-Alg_2_–PAM was confirmed by temperature-dependent experiments.

## 1. Introduction

As science and technology progressed, several new diseases emerged, necessitating the development of new antibiotics for their treatment. Although antibiotics have been beneficial to human health, excess antibiotics have become water pollutants, and studies are being conducted for their removal [1,2]. Ciprofloxacin (CPX) antibiotics, which were introduced in 1987, are used to treat bone disorders, arthritis, skin infections, respiratory infections, and other conditions. When abused, there are side effects, such as nausea, vomiting, and diarrhea, which can lead to muscle weakness [3,4]. As an excess of CPX causes water pollution, studies of the removal of CPX-contaminated wastewater have advanced [5].

Three-dimensional (3D), double-network (DN) composites are well-known for their high mechanical strength and enhanced adsorption capacity [6,7]. Moreover, introducing some functional groups into DN structures results in a better adsorbent [8]. Several DNs with multiplex materials have recently been developed to improve the adsorption capacity [9]. Among these materials, graphene oxide (GO) and alginate are widely used as 3D DN hydrogel adsorbents [10].

GO, a two-dimensional sheet, is a good candidate for use as an adsorbent because of its high specific surface area, thermal stability, and oxygen functional groups [11,12]. Despite this, because of its high dispersity, GO is difficult to separate from an aqueous solution following adsorption. This disadvantage can be overcome by creating beads from alginate. Alginate consists of a linear chain of (1–4)-linked β-d-mannuronic acid (M) and α-l-guluronic acid (G). It can be transformed into beads using an ionic crosslinking method, the bonding of Ca^2+^ ions with G by the “egg-box” formation [13,14]. To enhance the adsorption capacity of CPX, polyacrylamide (PAM) was functionalized to the surface of GO/Ca-Alg_2_ beads. PAM has amide bonds, indicating a strong complexation with antibiotics [15].

In this study, the final synthesized GO/Ca-Alg_2_–PAM beads are evaluated, and the adsorption efficiency of CPX by batch experiments (isothermal and thermal effect) and physicochemical properties are confirmed using field emission scanning electron microscopy (FE-SEM), scanning electron microscopy and energy dispersive X-ray spectroscopy (SEM-EDS), Fourier transform infrared spectroscopy (FT-IR), and a TGA analysis.

## 2. Materials and Methods

### 2.1. Materials

All the chemicals were analytical-grade reagents. Calcium chloride dihydrate (CaCl_2_·2H_2_O, 85%) and sodium alginate (Na-Alg) were purchased from Daejung Chemicals & Metals Co. (Kyungpook, Korea). GO and polyacrylamide (PAM) were purchased from Aldrich (Tokyo, Japan). A simulated solution was prepared using ciprofloxacin (CPX, C_17_H_18_FN_3_O_3_, 98%, Aldrich, Tokyo, Japan). All solutions were prepared with ultrapure water (18.2 MΩ·cm) from Vivagen Co., Ltd. (EXL5 Analysis 16, Daejeon, Korea).

### 2.2. Synthesis of GO/Ca-Alg_2_–PAM Beads

GO/Ca-Alg_2_–PAM adsorbents were synthesized by referring to the method by Fei et al. [16]. GO measuring 0.1 g was dispersed with 100 mL ultrapure water by sonication (JAC-4020P, KODO Technical Research Co., Hwaseong, Korea). Next, 0.6 g Na-Alg was added and stirred to ensure homogeneity. PAM powder measuring 1.0 g was slowly added and heated at 80 °C for 4 h. When the solution became sticky, it was allowed to cool at 30 °C. Thereafter, for making beads, the solution was dropped at 2 mL/min using a syringe pump in 10 g/L CaCl_2_⋅2H_2_O mixture. The GO/Ca-Alg_2_–PAM beads in CaCl_2_⋅2H_2_O solution were stirred for 24 h at room temperature to stabilize the beads. The resulting beads were washed three times with ultrapure water to remove impurities and dried at 80 °C in the oven.

### 2.3. Characterization of the GO/Ca-Alg_2_–PAM Beads

An FE-SEM was used to examine the morphology of GO/Ca-Alg_2_–PAM beads (SU8220, Hitachi, Japan). FT-IR (Frontier, PerkinElmer, Waltham, MA, USA) and TGA analyses (Q600, TA Instruments, New Castle, DE, USA) were used to determine the physicochemical properties of the GO/Ca-Alg_2_–PAM beads, including chemical functional combination and thermal properties.

### 2.4. Adsorption Experiments

All experiments were proceeded in a duplicate batch system using a 50 mL conical tube (PE, SPL Korea, Hwaseong, Korea). For adsorption isotherm experiments, CPX adsorption was performed at 250 rpm for 24 h at room temperature using solutions of various concentrations (1–50 ppm). The amount of GO/Ca-Alg_2_–PAM beads used at this time was 0.05 g, and the volume of the contact solution was 55 mL. The solution was separated from adsorbents by centrifugation at 3500 rpm for 10 min. The solution was filtered by 0.20 μm filters (Whatman, nitrocellulose membrane filters). The residual concentration of CPX was measured by a UV–visible spectrophotometer (Libra S60, Biochrom, Hwaseong, Korea) at 270 nm.

The equilibrium *q_e_* value equation is shown below:

(1) $$q_{e}=\frac{\left(C_{o}-C_{e}\right)V}{W}$$

where *q_e_* is the adsorption capacity (mg/g), and *C_o_* and *C_e_* are the before and after adsorption concentrations (ppm) of CPX, respectively. *V* represents the contact solution volume (mL) and *W* represents the weight of the adsorbent (g).

Temperature effect adsorption experiments with 0.5, 1, and 2 ppm CPX were performed at 10 °C, 25 °C, and 40 °C. The adsorption procedure was identical to the isothermal adsorption experiment.

## 3. Results

### 3.1. Characterization of GO/Ca-Alg_2_–PAM Beads

**Figure 1:**
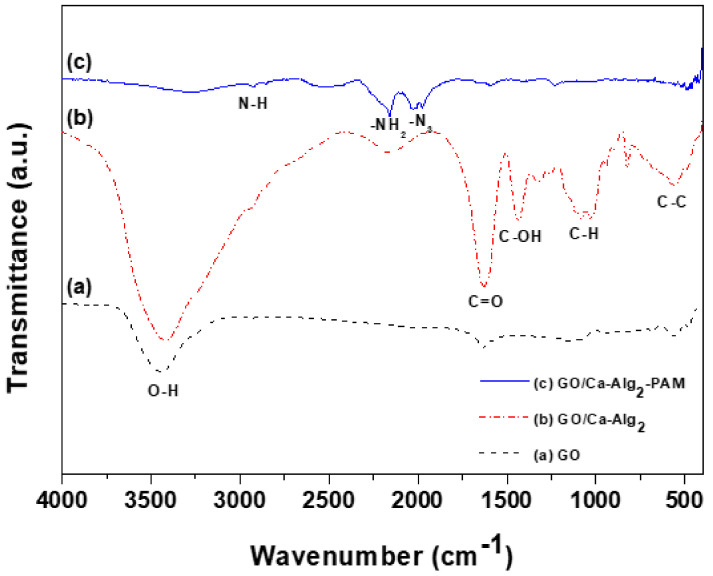
Fourier transform infrared spectra of (**a**) graphene oxide (GO), (**b**) GO/Ca-Alg_2_, and (**c**) GO/Ca-Alg_2_–PAM.

The FT-IR spectra of the GO, GO/Ca-Alg_2_, and GO/Ca-Alg_2_–PAM in the 4000–400 cm^−1^ region are showed in Figure 1. The new peaks of GO/Ca-Alg_2_ differed from GO at 1453, 1358, 1249, and 1074 cm^−1^. The peaks indicated the –COO stretching vibrations, C–H stretching bands at 1453 and 1074 cm^−1^, a C–OH bending band at 1357 cm^−1^, and a C–O–C asymmetric stretching band at 1249 cm^−1^. As a result, the DN composites were synthesized successfully as GO/Ca-Alg_2_ beads [17]. Figure 1 demonstrates the GO/Ca-Alg_2_–PAM peaks, functionalizing the PAM polymer, stretching the N-H peak at 2819 cm^−1^, –NH_2_ peak at 2161 cm^−1^, and –N_3_ peak at 2017 cm^−1^ [18]. However, the broad O-H peak at 3500–2500 cm^−1^ disappeared. Finally, GO/Ca-Alg_2_–PAM beads were successfully synthesized as 3D DN composites for removing CPX.

**Figure 2:**
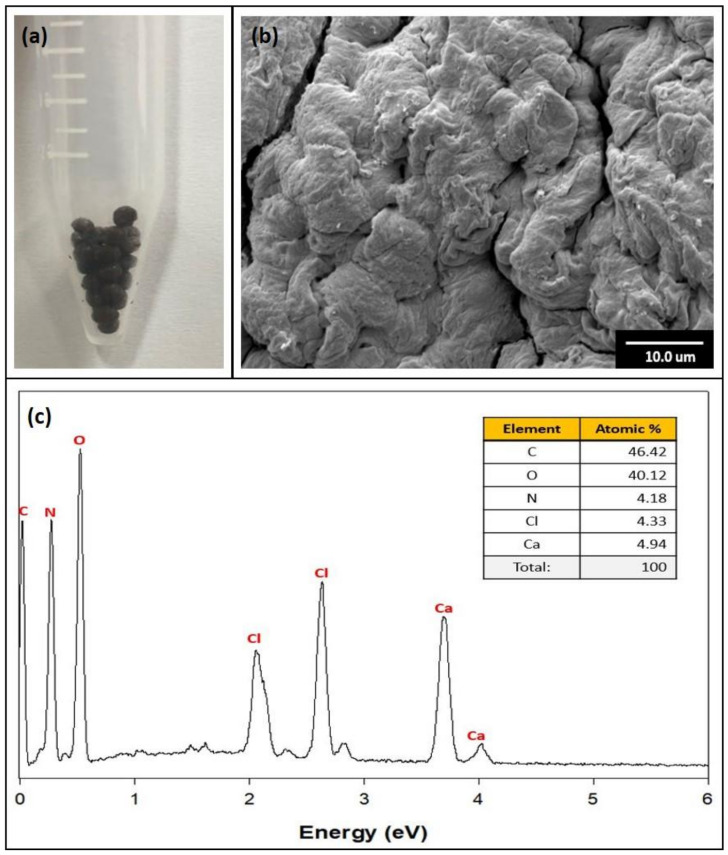
(**a**) GO/Ca-Alg_2_–PAM beads, (**b**) scanning electron microscopy image, and (**c**) scanning electron microscopy and energy dispersive X-ray spectroscopy spectra.

The image and SEM images of the GO/Ca-Alg_2_–PAM beads are shown in Figure 2a,b. The dark brown beads in the 3D DN composite were formed uniformly. The SEM analysis magnified the beads more than 1000 times, confirming that the combination of GO, alginate, and PAM formed a rugged surface. Each material, the GO or polymer, was not observed by the combination of the GO and polymer; the EDS elemental analysis confirmed that the synthesis was successful (Figure 2c). As a result, the spectra showed the peaks of C, O, N, Ca, and Cl. The GO was composed of C, O, and H. As the Na-Alg was converted into Ca-Alg_2_ by the beads, Ca and Cl peaks appeared. The PAM consisted of C, O, and N elements. Because H is not analyzed by SEM-EDS, it was excluded from the theoretical value, while its atomic percentage was calculated (anal. Calcd. for GO/Ca-Alg_2_–PAM: C, 46.42; O, 40.12; N, 4.18; Ca, 4.91; Cl, 4.33).

**Figure 3:**
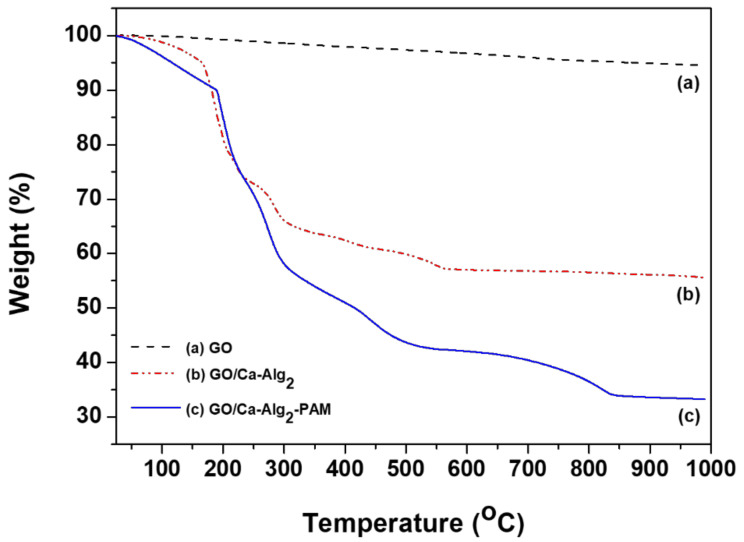
Thermogravimetric analysis curves of (**a**) graphene oxide, (**b**) GO/Ca-Alg_2_, and (**c**) GO/Ca-Alg_2_–PAM.

Figure 3 depicts the TGA estimation results of up to 1000 °C. Because of its high thermal stability, GO’s weight loss was minutely reduced. However, GO/Ca-Alg_2_ and GO/Ca-Alg_2_–PAM showed a significant decrease of up to 300 °C, with a weight loss of approximately 34.87% and 40.13%, respectively. After rapid decomposition, no further decrease in the weight of GO/Ca-Alg_2_ was observed at high temperature. In contrast, GO/Ca-Alg_2_–PAM gradually decreased in weight with increasing temperatures. However, because the procedure was performed within 100 °C, the removal of CPX was deemed irrelevant.

### 3.2. Adsorption Equilibrium Isotherms

The equilibrium isotherms Langmuir [19], Freundlich [20], and Temkin [21] model equations were used to fit the adsorption isotherm data. The Langmuir (2), Freundlich (3), and Temkin (4) equations were expressed as follows:

(2) $$q_{e}=\frac{q_{m}bC_{e}}{1+bC_{e}}$$

(3) $$q_{e}=K_{f}C_{e}^{1/n}$$

(4) $$q_{e}=\frac{RT}{B}\ln K_{T}C_{e}$$

where *q_m_* is the maximum adsorption capacity of adsorbent (mg/g), and *b* represents the Langmuir adsorption constant connecting with the free energy of the adsorption (L/mg). In the Freundlich isotherm model, *K_f_* ((mg/g) (L/mg)^1/*n*^) and *n* are constants of the isotherm. *K_T_* (L/g) and *R* (8.314 J/mol/K) are the gas constants, and *B* (L/mg) represents the isotherm constant in Equation (4).

The error analysis was evaluated by the correlation coefficient (*r^2^*) and chi-square (*χ^2^*) [19] value. The value of chi-square (*χ^2^*) was calculated using Equation (5).

(5) $$\chi^{2}=\sum \frac{{\left(q_{e,calc.}-q_{e,\;exp}\right)}^{2}}{q_{e,\;exp}}$$

Here, *q_e, calc,_* and *q_e, exp_* were indicated as experimental and calculated values, respectively.

**Figure 4:**
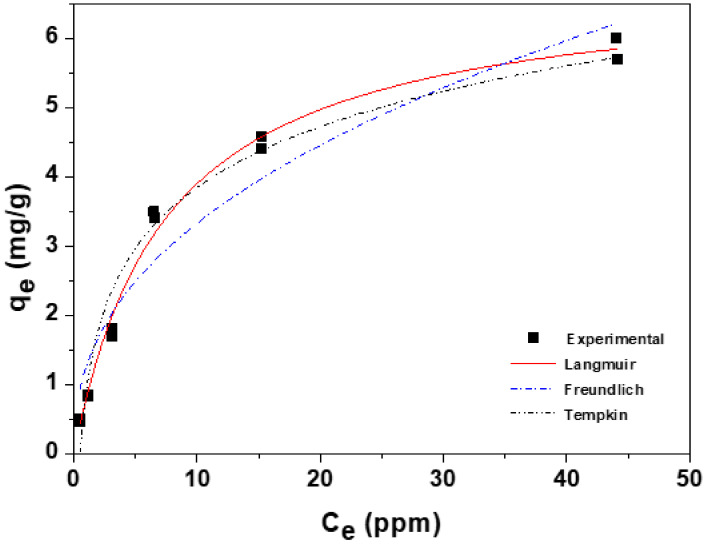
Adsorption isotherm and nonlinear fitting models of ciprofloxacin onto GO/Ca-Alg_2_–PAM beads.

The experimental data and fitted nonlinear lines were expressed using Origin 8.0 in Figure 4. The values of parameters, regression coefficients (*r^2^*), and chi-square (*χ^2^*) are given in Table 1. Among the isotherm models, the Langmuir isotherm was the best fitted with a maximum adsorption capacity of 6.846 mg/g. In the calculated error analysis, the values of *r^2^* and *χ^2^* were 0.991 and 0.037, respectively. To evaluate the efficiency of new GO/Ca-Alg_2_–PAM beads, a comparison of CPX *q_m_* values using various adsorbents is shown in Table 2. The *q_m_* value of GO/Ca-Alg_2_–PAM was neither very low nor exceptionally high compared to the existing literature. However, the adsorbents developed in the existing literature have a very fine powder form. Therefore, a separation process such as additional precipitation is required after the adsorption treatment. However, the GO/Ca-Alg_2_–PAM developed in this paper had a bead shape, so it is convenient to manage and has the advantage of an easy separation. In addition, when processing a large amount of waste liquid, the treatment is performed by filling the fixed-bed column, and the shape of the bead can be used efficiently by lowering the pressure drop.

### 3.3. Effect of Temperature and Thermodynamic Parameters

The effect of temperature on the CPX adsorption was estimated using thermodynamic parameters, including Gibb’s free energy (Δ*G*), enthalpy (Δ*H*), and entropy (Δ*S*). The parameters of thermodynamics were calculated using the following equations from the Arrhenius equation [25]

(6) $$\Delta G=-\mathrm{RT}\ln(K_{c})$$

(7) $$\ln(K_{c})=\frac{\Delta S}{R}-\frac{\Delta H}{RT}$$

where *K_c_* is the equilibrium constant (*q_e_/c_e_*) (L/g), *R* is the ideal gas constant (8.314 J/mol∙K), and T (*K*) is the temperature of adsorption.

**Figure 5:**
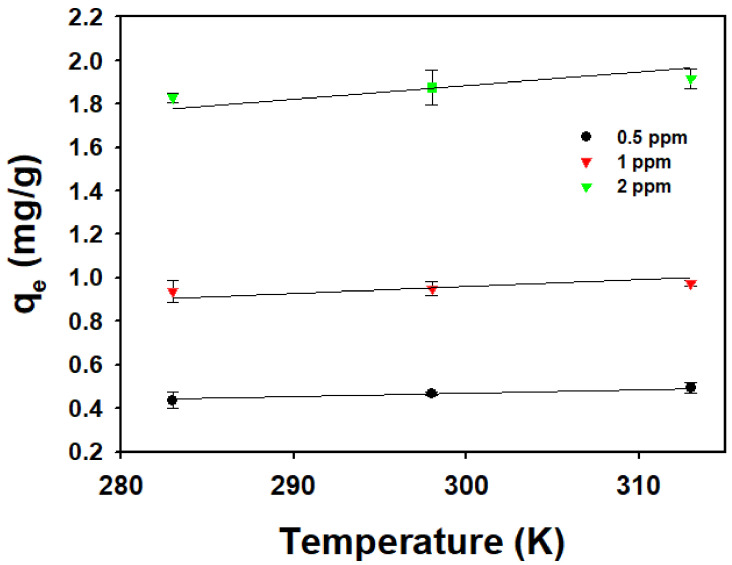
Ciprofloxacin adsorption onto GO/Ca-Alg_2_–PAM beads depending on temperature.

**Figure 6:**
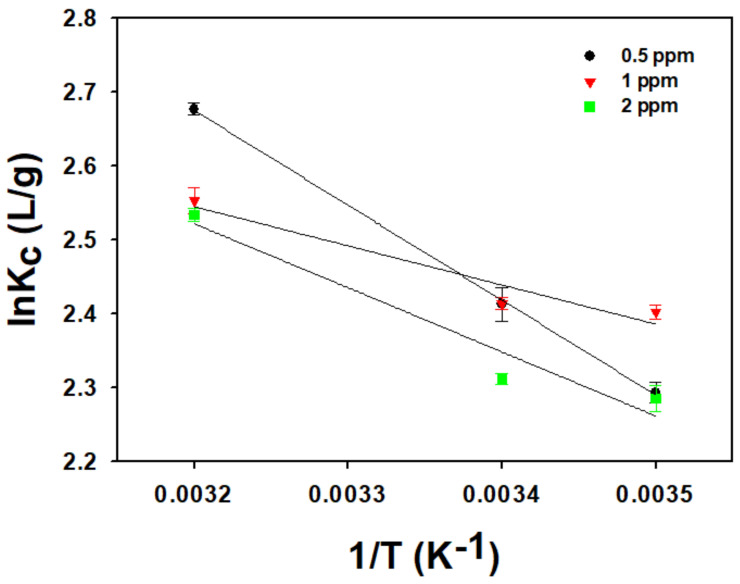
The plots of ln(*Kc*) vs. 1/T for ciprofloxacin adsorption on to GO/Ca-Alg_2_–PAM at 283, 298, and 313 K.

The adsorption equilibrium depending on temperature is indicated in Figure 5. All experiments of the temperature effect were conducted in triplicate. It was shown that the *q_e_* value increased with an increasing temperature in all concentrations of CPX. The thermodynamic parameters in Table 3 were evaluated from the slope and intercept of the van ’t Hoff plots of ln(*K_c_*) versus 1/T (Figure 6). The Δ*H* values ranged from 4.4 to 10.7 kJ/mol (mean value 7.43 kJ/mol). The entropy value of adsorption was calculated to be in the range of 35–56 J/mol∙K. To confirm that the adsorption was an endothermic system, the Gibbs free energy (Δ*G*), which indicates the spontaneity of the adsorption, was evaluated. The Δ*G* value indicated a spontaneous reaction with a negative value [26]. In calculation results, the negative values of Δ*G* were observed in all ranges, indicating that the adsorption was a spontaneous reaction in this study.

## 4. Conclusions

The GO/Ca-Alg_2_–PAM beads were successfully synthesized for the removal of CPX. Not only could the forming beads easily separate from the aqueous solution, but they could also weigh beads for adsorption. According to the Langmuir isotherm model, which best fit the experimental data, the 3D DN beads had a high CPX adsorption capacity of 6.846 mg/g. In experiments on the effect of temperature, the adsorption behavior showed a spontaneous response and had a high adsorption capacity at high temperature. Furthermore, GO/Ca-Alg_2_–PAM beads had a stability of up to 100 °C, which is sufficient for use in actual wastewater treatment. Finally, the new adsorbent, GO/Ca-Alg_2_–PAM beads, is an excellent material for removing CPX from an aqueous solution.

## Figures and Tables

**Table 1:** Parameters, regression coefficients (*r^2^*), and chi-square (*χ^2^*) of ciprofloxacin adsorption isotherm models using GO/Ca-Alg_2_–PAM beads.

Isotherm Model	Parameters	*r^2^*	*χ^2^*
Langmuir	*q_m_* = 6.846 mg/g *b* = 0.132 L/mg	0.991	0.037
Freundlich	*K_F_* = 1.260 (mg/g)(L/mg)^1/*n*^ *n* = 2.372	0.932	0.282
Temkin	*B* = 1954.69 L/mg *K_T_* = 2.074 L/g	0.966	0.142

**Table 2:** Comparison of ciprofloxacin maximum adsorption capacity between this study and previous studies.

Adsorbents	*q_m_* (mg/g)	Temperature (K)	References
GO/Ca-Alg_2_–PAM	6.846	298	This study
MCFA	1.547	313	[20]
GAC	217.4	298	[21]
Kaolinite	7.952	308	[22]
Goethite	19.88	295	[23]
ZnO-BC-2-650	0.449	298	[24]

**Table 3:** The thermodynamic parameters of ciprofloxacin adsorption on to GO/Ca-Alg_2_–PAM.

CPX (ppm)	Δ*H* (kJ/mol)	Δ*S* (kJ/mol∙K)	Δ*G* (kJ/mol)		
			283 K	298 K	313 K
0.5	10.7	0.056	−5.29	−6.13	−6.98
1	4.4	0.035	−5.57	−6.10	−6.63
2	7.2	0.044	−5.25	−5.91	−6.57
